# NMR structure of the *Bacillus cereus* hemolysin II C-terminal domain reveals a novel fold

**DOI:** 10.1038/s41598-017-02917-4

**Published:** 2017-06-12

**Authors:** Anne R. Kaplan, Katherine Kaus, Swastik De, Rich Olson, Andrei T. Alexandrescu

**Affiliations:** 1 Department of Molecular and Cell Biology, University of Connecticut, 91 N. Eagleville Rd, Storrs, CT 06269-3125 USA; 2 Department of Molecular Biology and Biochemistry, Molecular Biophysics Program, Wesleyan University, 224 Hall-Atwater, 52 Lawn Ave., Middletown, CT 06459-0175 USA; 3 Department of Molecular Biophysics and Biochemistry, Yale University, 266 Whitney Avenue, New Haven, CT 06520-8114 USA

## Abstract

In addition to multiple virulence factors, *Bacillus cereus* a pathogen that causes food poisoning and life-threatening wound infections, secretes the pore-forming toxin hemolysin II (HlyII). The HlyII toxin has a unique 94 amino acid C-terminal domain (HlyIIC). HlyIIC exhibits splitting of NMR resonances due to *cis/trans* isomerization of a single proline near the C-terminus. To overcome heterogeneity, we solved the structure of P405M-HlyIIC, a mutant that exclusively stabilizes the *trans* state. The NMR structure of HlyIIC reveals a novel fold, consisting of two subdomains αA-β1-β2 and β3-β4-αB-β5, that come together in a barrel-like structure. The barrel core is fastened by three layers of hydrophobic residues. The barrel end opposite the HlyIIC-core has a positively charged surface, that by binding negatively charged moieties on cellular membranes, may play a role in target-cell surface recognition or stabilization of the heptameric pore complex. In the WT domain, dynamic flexibility occurs at the N-terminus and the first α-helix that connects the HlyIIC domain to the HlyII-core structure. In the destabilizing P405M mutant, increased flexibility is evident throughout the first subdomain, suggesting that the HlyIIC structure may have arisen through gene fusion.

## Introduction

The soil-dwelling, spore-forming *B*. *cereus* bacterium^1–3^ produces a number of virulence factors^4^ including several secreted pore-forming toxins (PFTs) that form lytic channels in the membranes of target cells^5^. One of these toxins, hemolysin II (HlyII), is present in several closely related Bacillus species including *B*. *cereus*, *B*. *thuringiensis* (a bacterium that parasitizes insects and has insecticide applications), and *B*. *anthracis* (the cause of anthrax)^6, 7^. In *B*. *cereus*, expression of HlyII is under the control of the HlyIIR protein^8^, and the Fur system that regulates iron homeostasis^9, 10^. Expression of HlyII is greater under oxic conditions than under conditions mimicking the intestinal tract, suggesting the toxin may not play a major role in gastrointestinal disease^11^. Although the physiological target of HlyII is not known, the purified toxin lyses rabbit and human erythrocytes^12^ as well as other cultured mammalian cells^13, 14^. In addition, the toxin can attack species like algae^15^ and insects. Studies in mice and insects suggest that HlyII is involved in virulence, and that the toxin causes apoptosis of macrophages *in vitro* and *in vivo* ^16^. HlyII belongs to a larger family of secreted toxins with similar predicted core structures including the *B*. *cereus* cytotoxin K (CytK)^17^, *Staphylococcal* hemolysins/leukocidins^18, 19^, and toxins secreted by a variety of *Vibrio* species^20^. Similar to other family members, HlyII is secreted as a water-soluble monomer that assembles into a heptameric pore following binding to cell membranes^12, 21^. A unique feature of HlyII is the attachment of a C-terminal domain consisting of 94 amino acids that shows no sequence or structural homology to other known proteins^18^. The C-terminal domain, henceforth referred to as HlyIIC, is the subject of the present study.

Removal of the HlyIIC domain reduces the activity of HlyII against rabbit erythrocytes 8-fold^12^, but the mechanism by which this domain affects PFT activity is unknown. Homologs of HlyII from Staphylococcal PFTs do not contain C-terminal extensions, but homologous toxins from *Vibrio* species have C-terminal domains with lectin-folds that bind glycan receptors on target cell surfaces^22, 23^. Since HlyIIC occupies a similar topological position in HlyII, it could act as a targeting domain against cell-surface receptors to aid binding to target membranes, analogous to the C-terminal extensions of *Vibrio* PFTs. These C-terminal lectin domains, however, have no sequence homology to HlyIIC and unrelated β-trefoil and β-prism structures^24^.

To investigate the HlyIIC domain, we expressed, purified, and solved the structure of the domain using solution-state nuclear magnetic resonance (NMR). The wild-type HlyIIC domain (WT-HlyIIC) exists in two states due to *cis/trans* isomerization of the single proline in the sequence at position 405 (here the HlyIIC domain is numbered according to the full length HlyII toxin). To reduce the spectral complexity arising from separate sets of NMR resonances for the *cis* and *trans* conformations^25^, we solved the structure of a P405M mutant of HlyIIC which eliminates the *cis* conformational state. The NMR structure of the HlyIIC domain reveals a novel, previously undescribed fold. In addition to structural studies, we characterized the backbone dynamics of the domain using NMR relaxation experiments and hydrogen exchange. To gain functional insights we modeled the domain in the context of the full-length HlyII protein. The NMR studies define the structural and dynamic properties of the HlyIIC domain and serve as a starting point for understanding its possible functions.

## Results

### The structure of HlyIIC reveals a novel fold

**Figure 1:**
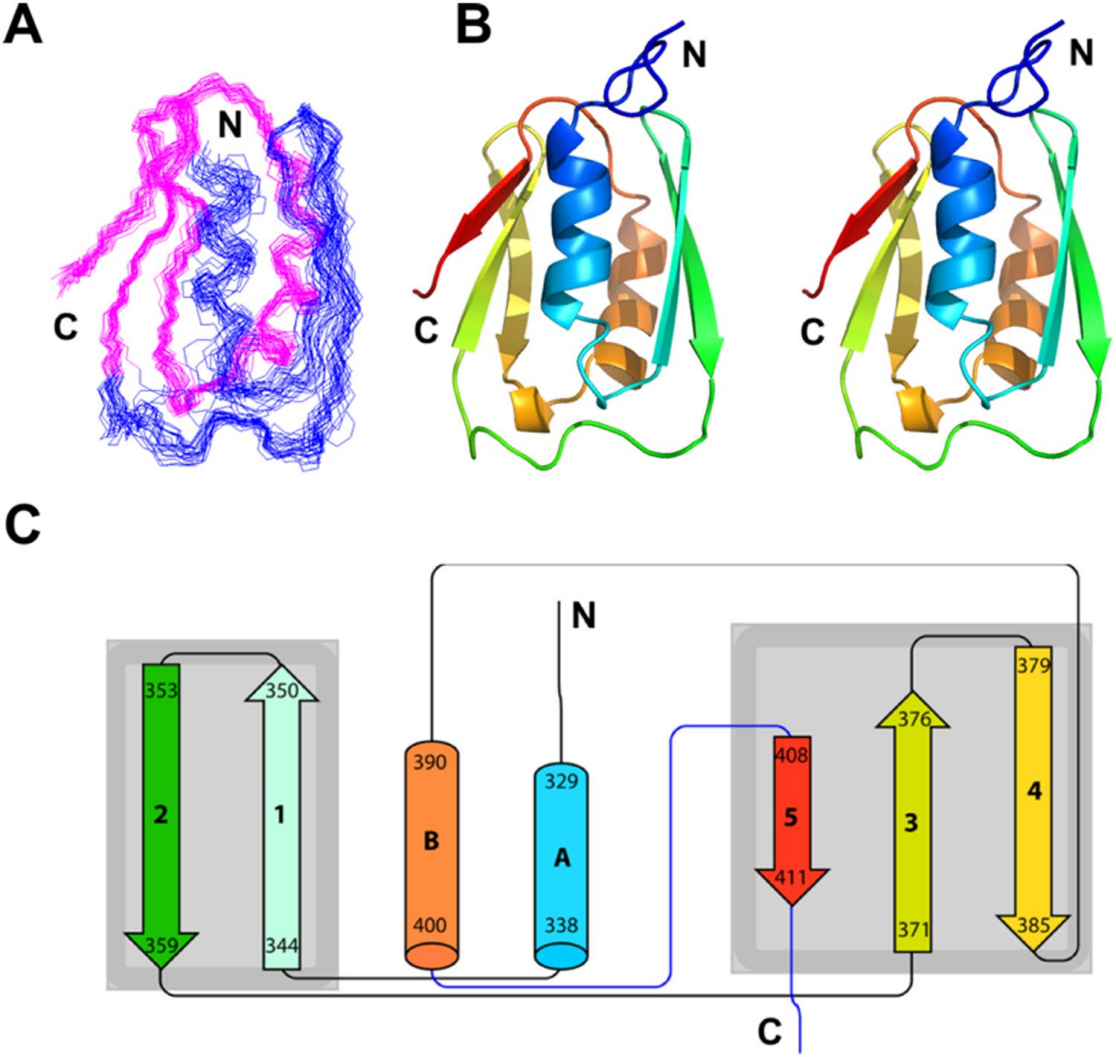
NMR structure of HlyIIC. (**A**) Best-fit superposition for the backbone structures of residues 330–412 from the ensemble of 25 lowest energy NMR structures. The first 11 residues at the disordered N-terminus are not shown. The N- and C-terminal halves of the molecule are colored blue and magenta, respectively, to illustrate the better precision for the 370–412 segment in subdomain 2 (magenta). (**B**) Stereo diagram (‘wall-eyed’ view) of the NMR structure closest to the ensemble average. The structure is colored on a gradient running from blue (N-terminus) to red (C-terminus). The view is the same as in (**A**). (**C**) Diagram summarizing the folding topology of the structure. Cylinders depict α-helices and arrows indicate β-strands. Secondary structure elements are labeled according to their position in the sequence, together with their start and end residues. The coloring scheme is the same as in (**B**). Additional elements of secondary structure not shown in the figure, are a 3_10_ helix between residues E386-T389 which is present in most of the NMR structures, and a 3_10_ helix between N320-L324 that occurs at the disordered N-terminus of only some of the structures in the NMR ensemble.

The NMR structure ensemble of the P405M-HlyIIC mutant is shown in Fig. 1A. Statistics for the NMR structure are given in Table 1. HlyIIC folds into an α + β architecture, consisting of two α-helices and five strands of anti-parallel β-sheet (Fig. 1B). The overall topology and secondary structure limits are summarized in Fig. 1C. The structure can be thought of as consisting of two subdomains. The first subdomain is comprised of the N-terminal α-helix αA and a β-hairpin: αA-β1-β2 (blue in Fig. 1A). The second subdomain has the last three β-strands with a non-sequential anti-parallel pairing between strands β3 and β5, and an α-helix intervening between strands β4 and β5: β3-β4-αB-β5 (magenta in Fig. 1A).

**Table 1:** Statistics for the 25 Lowest Energy NMR Structures of HlyIIC.

**Experimental Restraints**		
NMR Restraints (total)^*a*^	1790	
Distance (total)	1592	
Intraresidue NOEs	690	
Sequential NOEs	417	
Short range NOEs (1 < |i-j| < 5)	160	
Long range NOEs (5 ≤ |i-j|)	271	
Hydrogen bonds (27 × 2)	54	
Dihedral (ϕ 80, ψ 80, χ_1_ 38)	198	
**Residual restraint violations** ^***b***^		
NOE (Å)	0.040 ± 0.002	
Dihedral (°)	0.42 ± 0.09	
**RMSD from ideal geometry**		
Bonds (Å)	0.0050 ± 0.0002	
Angles (°)	0.66 ± 0.02	
Improper torsions (°)	1.900 ± 0.002	
Ramachandran statistics^*c*^		
most favored (%)	80	
allowed (%)	16	
generously allowed (%)	5	
disallowed (%)	0	
ProcheckNMR Z-score^*c*^	−4.55	
Molprobity clash score^*c*^	−3.98	
**Coordinate RMSD (Å)**		
NMR ensemble to average	Backbone^*d*^	All Heavy
Entire domain (94 a.a.)	1.86 ± 0.46	2.26 ± 0.46
Excluding first 11 a.a.: 330–412 (83 a.a.)	0.96 ± 0.21	1.38 ± 0.21
Regular secondary Structure (51 a.a.)^*e*^	0.88 ± 0.19	1.18 ± 0.24
β-sheet only (31 a.a.)^*e*^	0.72 ± 0.19	1.07 ± 0.35
Subdomain 1: 330–360 (31 a.a.)	1.04 ± 0.26	1.52 ± 0.26
Subdomain 2: 370–412 (43 a.a.)	0.54 ± 0.18	0.93 ± 0.18

^*a*^Grouping of non-redundant restraints was performed using the program QUEEN (http://www.cmbi.ru.nl/software/queen)^58^.

^*b*^Structures have no NOE violations greater than 0.3 Å or dihedral angle violations more than 5°.

^*c*^Calculated using the Protein Structure Validation Suite (http://psvs-1_5-dev.nesg.org)^59^.

^*d*^Calculated using C′, N, and Cα backbone atoms.

^*e*^The regular secondary structure of the HlyIIC domain is 330–338 (αA), 344–350 (β1), 353–359 (β2), 371–376 (β3), 379–385 (β4), 390–400 (αB), 408–411 (β5).

To investigate structure similarity relationships for HlyIIC we performed a structural homology search using the Dali server^26^. The search failed to identify homologs, with the closest hit having a Z-score of 1.8 that is not considered significant^26^. The closest hit was a portion of formyltetrahydrofolate deformylase (PDB code 3NRB), which gave an RMSD of 3.0 Å to HlyIIC. Inspection of this structure, however, showed that the two proteins do not share the same topology. We thus conclude that HlyIIC represents a previously uncharacterized, novel protein fold.

### Hydrophobic core and electrostatic surface properties of the structure

**Figure 2:**
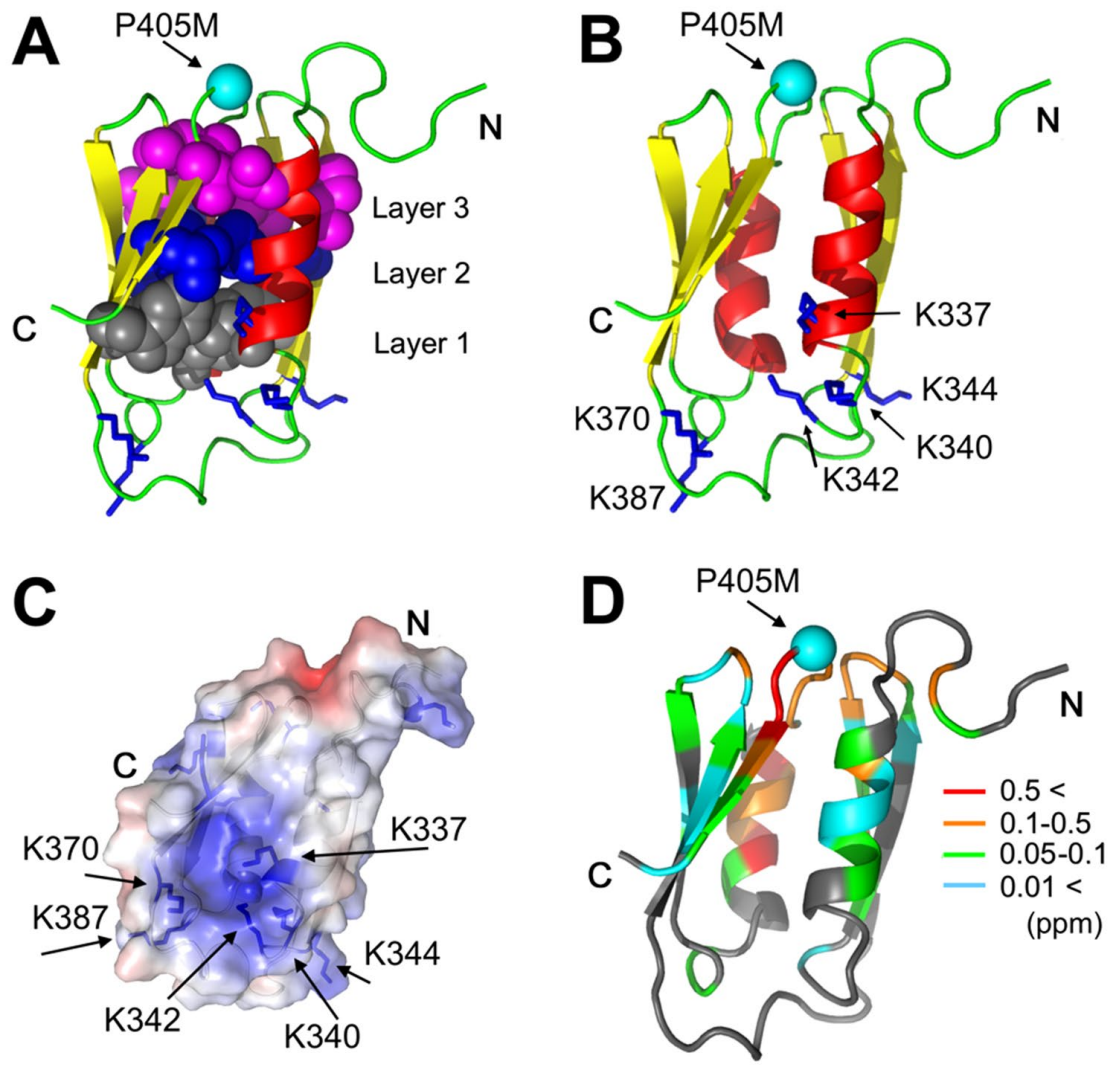
Structural properties of the HlyIIC domain. (**A**) Cartoon of the HlyIIC NMR structure closest to the ensemble average, illustrating the 3-layer hydrophobic core of HlyIIC. (**B**) The relationship between P405, which is subject to *cis/trans* isomerization in WT-HlyIIC and a patch of positively charged lysine residues at the bottom of the structure. (**C**) Electrostatic surface of HlyIIC calculated using the APBS method^32^, showing the positively charged patch formed by basic lysine residues. (**D**) Structural mapping of chemical shift differences between the conformational states of WT-HlyIIC related by *cis/trans* isomerization of P405. The composite (|**∆**HN| + 0.1|**∆**N|) chemical shift index data^25^ are colored on a gradient running from cyan for the smallest differences to red for the largest. Residues that do not show splitting of ^1^H-^15^N resonances due to *cis*/*trans* isomerization are in gray.

Figure 2 shows some of the key side-chain interactions and surface properties of the structure. The topology of HlyIIC is unusual in that it combines α-helices and β-strands in the same layer - an arrangement thought to be disfavored since α-helices cannot hydrogen bond to β-strands^27^. Consistently, there are no main-chain hydrogen bonds between the two subdomains in the NMR structure, and we did not detect through H-bond couplings between the two subdomains in the lrHNCO (long-range HNCO)^28^ experiment. Rather, the two β-sheets are flanked on either side by helices αA and αB, and come together through hydrophobic contacts to form a barrel-like structure with pseudo-two-fold symmetry. Similar to 5-stranded anti-parallel Greek-key β-barrels seen in examples such as the OB-fold^29–31^, the hydrophobic core of the structure is arranged into three layers of non-polar residues (Figs 2A and S1A). The non-polar residues that make up the hydrophobic core are arranged with a 2-residue sequence periodicity in the β-strands and with a 3 to 4 residue periodicity in the α-helices (Fig. S1A). Representative NMR data illustrating NOESY distance contacts for core hydrophobic residues are shown in Fig. S2. Excluding the disordered first 11 amino acids, only five of 32 non-polar residues in the HlyIIC structure do not participate in the three-layer hydrophobic core structure and are surface exposed. Three of these, Y363, I365, Y367 are in the extended loop between strands β2 and β3 that connects subdomains 1 and 2 in the polypeptide chain. Residues F375 from β3 and Y406, are surface-exposed and close in the structure to the site of the *cis/trans* peptide bond isomerization, G404-P405.

The HlyIIC domain is decorated by a number of charged residues, including 12 lysine, 6 glutamate, and 4 aspartate residues and has a calculated pI of 8.8. An electrostatic map computed using the APBS method^32^ indicates a predominantly basic surface, with a positive patch located on the side of the domain opposite the N-terminal connection to the rest of the HlyII toxin. This positive patch, on the side of the molecule we will refer to as the ‘bottom’, is formed by a sequence consisting of the residues 337-KLNKGKGKL-345, together with K370 and K387 (Fig. 2B,C).

### Sensitivity of the WT-HlyIIC structure to *cis/trans* isomerization of P405

The site of *cis*/*trans* peptide bond isomerization, G404-P405, which leads to conformational heterogeneity in NMR spectra of WT-HlyIIC is located at the top of the structure (Fig. 2D) near the N-terminal linker that connects HlyIIC to the core of the HlyII protein. We previously noted that *cis*/*trans* proline isomerization leads to the splitting of about half of the backbone crosspeaks in the ^1^H-^15^N HSQC spectrum of WT-HlyIIC^25^. With the structure of the HlyIIC domain, we can now map the extent to which the magnetic environments of amide protons are affected by *cis/trans* isomerization of the G404-P405 peptide bond (Fig. 2D). The largest composite ^1^H-^15^N chemical shift differences (red in Fig. 2D) are near P405 but also extend into the secondary structure elements flanking the proline-harboring loop, αB and β5. Intermediate perturbations (orange in Fig. 2D) occur in the loops between β1-β2 and β3-β4, which are close in space but not in the sequence to P405. Smaller composite chemical shift differences of 0.01 to 0.1 ppm (blue and green in Fig. 2D) extend throughout the entire barrel-like fold of the domain. The three-layer hydrophobic core of HlyIIC (Fig. 2A) may provide a conduit to communicate the P405 isomerization state to the rest of the structure.

### CD spectroscopy of the HlyIIC domain

**Figure 3:**
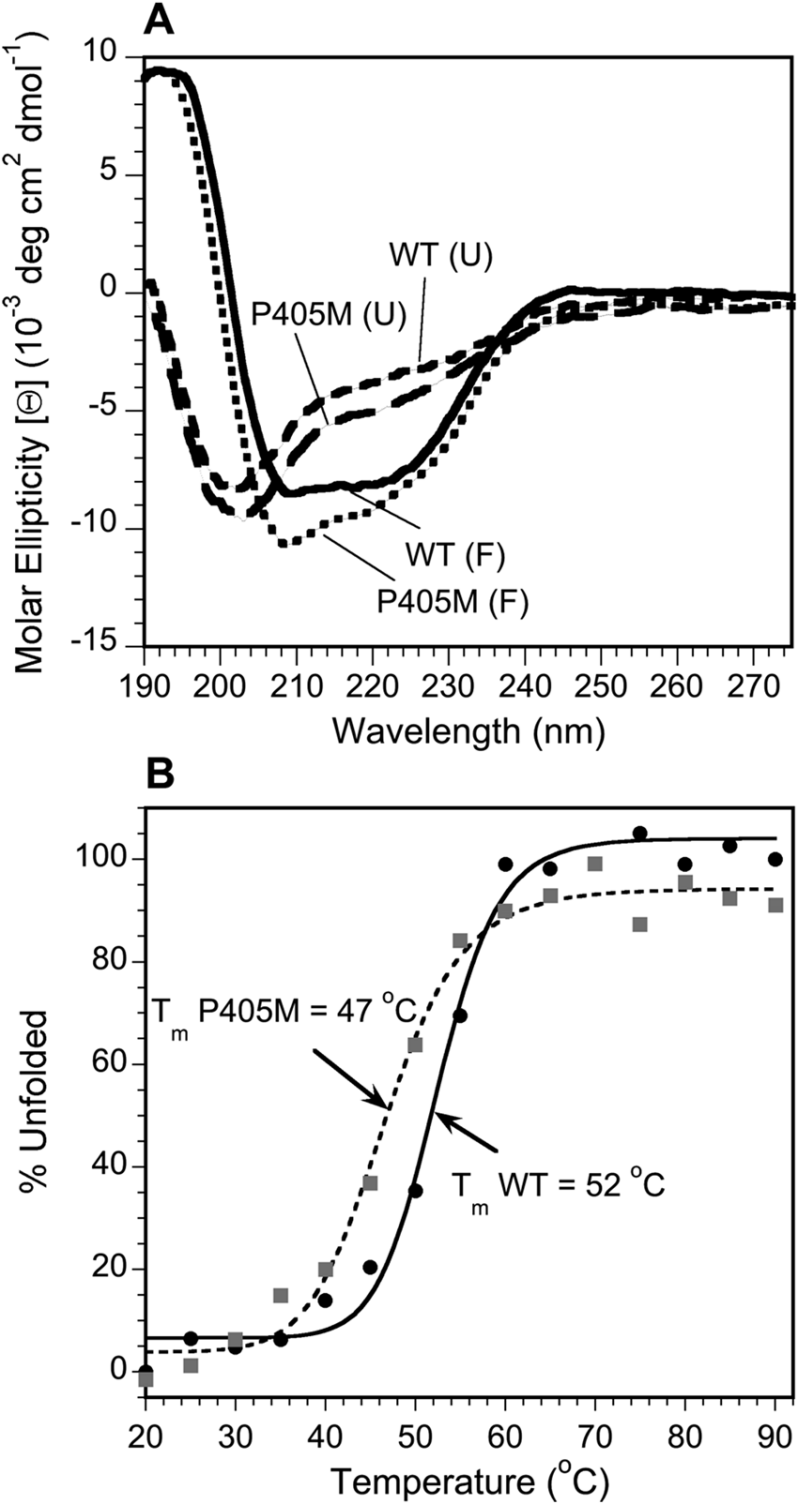
CD spectroscopy of HlyIIC. (**A**) Wavelength scans for folded WT-HlyIIC (solid line) and P405M-HlyIIC (dotted line) at 20 °C, together with thermally unfolded WT-HlyIIC (short dash) and P405M-HlyIIC (long dash) at 90 °C. (**B**) Thermal unfolding of WT-HlyIIC (filled circles, solid line) and P405M-HlyIIC (gray squares, dotted line).

To determine the extent to which the P405M mutation perturbs the HlyIIC domain, we compared CD data for the WT and mutant (Fig. 3). The folded-state CD spectra for WT-HlyIIC and P405M at 20 °C are very similar and typical of folded proteins with a mixture of α-helix and β-sheet structure (Fig. 3A). For comparison, the spectra at 90 °C (dashed lines in Fig. 3A) are typical of unfolded proteins, and very different from the folded state spectra at 20 °C. The CD spectra together with the conservation of chemical shifts between WT-HlyIIC and the P405M-HlyIIC mutant^25^ indicate that the overall folding topology is conserved. Structural differences may occur in the loops surrounding P405, the site of the P405M mutation. The extent of these structural differences will have to await the NMR structure determination of the *cis* and *trans* forms of WT-HlyIIC. Based on the observations above, however, the P405M-HlyIIC mutant is a suitable model for the overall WT-HlyIIC structure.

Figure 3B compares thermal unfolding data for WT-HlyIIC and the P405M-HlyIIC mutant monitored by ellipticity at 220 nm. The P405M mutation, destabilizes the protein to thermal unfolding by a modest 5 °C compared to the WT. As described below, NMR hydrogen exchange data indicate a subtler effect of the mutation, that involves the uncoupling of the dynamics of subdomain 1 from 2. The P405M-HlyIIC mutant was selected because a bulky methionine should most favor a *trans* peptide bond^33^. However, P405A-HlyIIC with a smaller alanine substitution, has a similar ^1^H-^15^N HSQC spectrum indicating a conserved structure, similar circular dichroism (CD) spectra, and a similar stability to thermal unfolding within 1 degree for that of P405M-HlyIIC (Fig. S3).

### Backbone dynamics indicate higher flexibility for the N-terminal subdomain

**Figure 4:**
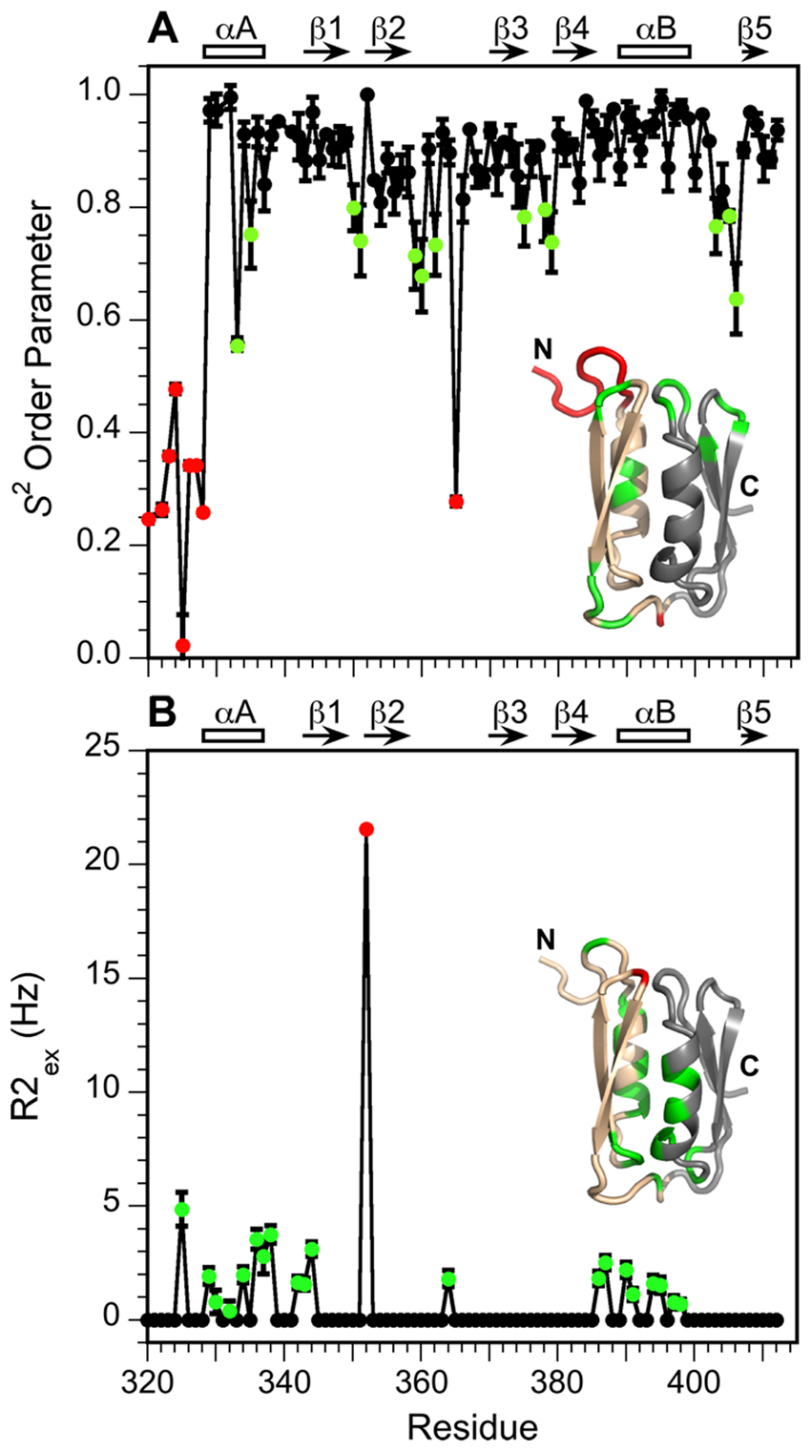
Backbone dynamics of the HlyIIC domain. (**A**) *S* ^2^ order parameters describing the amplitudes of ^1^H-^15^N bond motions on the ps-ns timescale. *S* ^2^ values smaller than 0.8 are shown in green and those smaller than 0.5 in red. (**B**) R2_ex_ line-broadening contributions to ^15^N R2 relaxation due to conformational averaging on the µs-ms timescale. R2_ex_ values greater than 0 Hz are shown in green and those greater than 5 Hz in red. The secondary structure of HlyIIC is shown at the top of panels. Error bars are shown for all values but in some cases are smaller than the symbols used to depict the data. The insets show the *S* ^2^ and R2_ex_ values mapped on the HlyIIC structure, with the first half of the molecule colored peach and the second half gray.

As shown in Fig. 1A and Table 1, the NMR structure of the C-terminal subdomain 2 (magenta) is more precisely determined than that of the N-terminal subdomain 1 (blue). Recall that the two subdomains are not linked through backbone hydrogen-bonds but come together solely through side-chain interactions. The differences in structural precision of the two segments could be due to differences in dynamics, or an artifact of a lower number of NMR restraints for the N-terminal half of the molecule (the distribution of long-range NOEs and H-bonds is 171 within subdomain 2, compared to 100 within subdomain 1, and 29 between the two subdomains). All aromatic residues in HlyIIC occur in the C-terminal subdomain 2. Aromatic residues in proteins typically improve spectral dispersion as well as contributing NOE distance contacts due to their hydrophobic nature, factors that may lead to the higher precision of the structure in subdomain 2. Alternatively, subdomain 1 may have intrinsically greater flexibility. To distinguish between these possibilities, we collected ^15^N NMR relaxation data on the backbone dynamics of P405M-HlyIIC (Fig. S4). The ^15^N relaxation data for P405M were analyzed with the Model-Free formalism^34^ to obtain *S* ^2^ order parameters that describe the amplitudes of backbone ^1^H-^15^N bond librations on the ps-ns timescale (Fig. 4A) and R2_ex_ contributions to transverse relaxation that are sensitive to conformational exchange processes on the µs-ms timescale (Fig. 4B).

The *S* ^2^ order parameter ranges from a value of one for rigid sites to zero for sites that have unhindered motion^34^. As shown in Fig. 4A, most residues in the regular secondary structure HlyIIC have *S* ^2^ order parameters in the rigid limit. Low *S* ^2^ order parameters indicative of dynamic flexibility occur for the N-terminal segment that connects HlyIIC to the rest of the HlyII protein, the N-terminal α-helix (αA), and the loops between secondary structure units - in particular the loop between helix αB and strand β5 that includes the site of the P405M mutation. The dynamic nature of the N-terminal linker segment corresponding to the first 11 amino acids of HlyIIC appears to be conserved in the full-length HlyII toxin, in as much as HlyIIC can be proteolytically cleaved from HlyII^12, 21^. The mean *S* ^2^ order parameters for residues 330–360 in subdomain 1 and 370–412 in subdomain 2 are near 0.9, and statistically indistinguishable. Therefore, it does not appear that the lower precision of subdomain 1 in the NMR structure is due to increased dynamics of this segment on the ps-ns timescale. The exception is helix αA, which has residues with moderately lower *S* ^2^ order parameters in an element of regular secondary structure that may contribute to the poorer definition of subdomain 1 in the NMR structure.

Figure 4B summarizes the sequence distribution of residues with R2_ex_ line-broadening contributions to the transverse relaxation rate, R2. The non-zero R2_ex_ terms are due to conformational exchange on the µs-ms timescale. The majority of residues that experience R2_ex_ contributions are in the two helices αA and αB, with some additional ones in the loops connecting elements of regular secondary structure. By contrast, R2_ex_ terms are not seen for the β-strands (Fig. 4B). These observations suggest that the α-helices and loops are involved in some type of conformational exchange process on the µs-ps timescale, that does not affect the β-sheets. A greater fraction of residues in subdomain 1 (36%) than subdomain 2 (19%) show conformational-exchange line broadening contributions, and the R2_ex_ terms are somewhat larger for subdomain 1 in the first half of the protein (Fig. 4B). The lower precision of subdomain 1 in the NMR structure is thus likely to be related to its greater flexibility on the µs-ms timescale rather than on the faster ps-ns timescale.

### Hydrogen exchange points to enhanced dynamics for the N-terminal subdomain in the P405M mutant

**Figure 5:**
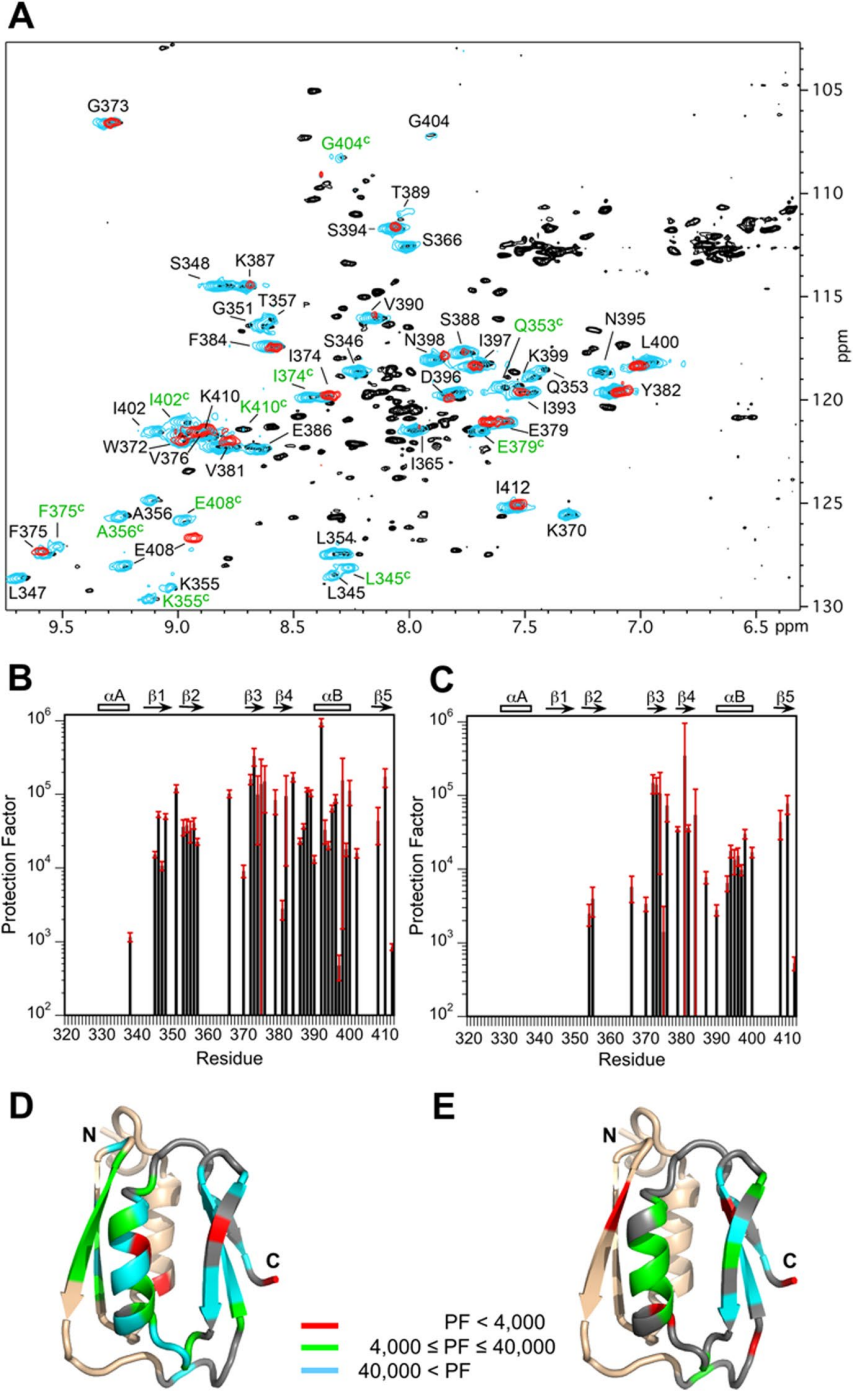
Hydrogen exchange of HlyIIC. (**A**) Superposition of the ^1^H-^15^N HSQC spectrum of the WT-HlyIIC domain in H_2_O (black) with those of WT-HlyIIC (cyan) and the P405M-HlyIIC mutant (red) after 2 h of incubation in D_2_O. Amide protons protected after 2 h of exchange in D_2_O are labeled (the full assignments for WT-HlyIIC and P405M-HlyIIC are published^25^). Protected amide protons from the *cis* form of WT-HlyIIC are labeled with green letters, these signals are missing in the P405M mutant, which eliminates the *cis* conformational form. E408 has a large chemical shift difference between the WT and the mutant because it is close to the site of the P405M mutation. Note that compared to WT-HlyIIC, only amides from the second half of the domain, residues 372–410, persist for 2 h in D_2_O for the P405M-HlyIIC mutant. Protection factors for (**B**) WT-HlyIIC and (**C**) P405M-HlyIIC. Protection factors are only given for amide protons from the *trans* form of the WT protein. Additional protection is seen for the side-chain Nε1 proton of W372, which exchanges with rates of 0.0029 ± 0.0006 min^−1^ and 0.106 ± 0.008 min^−1^ in WT-HlyIIC and P405M-HlyIIC, corresponding^57^ to protection factors of about 17,000 and 500 at pH 6. Protection factors for (**D**) WT-HlyIIC and (**C**) P405M-HlyIIC, mapped on the NMR structure of P405M-HlyIIC with the indicated scale.

Figure 5 compares hydrogen exchange (HX) data for WT-HlyIIC and P405M-HlyIIC. In Fig. 5A, a control ^1^H-^15^N HSQC of WT-HlyIIC in H_2_O (black) is superimposed with those obtained after 2 h of exchange in D_2_O for WT-HlyIIC (cyan) and P405M-HlyIIC (red). About 40 of the 92 backbone amide protons in WT-HlyIIC persist after 2 h in D_2_O (cyan peaks in Fig. 5A). These protected amide protons are distributed throughout the regular secondary structure of the HlyIIC domain. By contrast in the P405M-HlyIIC mutant, only amide protons from residues in subdomain 2 survive after 2 h in D_2_O. The result suggests that HX from subdomain 1 is selectively enhanced in the P405M-HlyIIC mutant.

Protection factors that describe the fold-decrease in HX rates due to structure compared to the chemically-determined intrinsic HX rates^35^, are given for WT-HlyIIC and P405M-HlyIIC in Fig. 5B and C, respectively. There are ~10 protected backbone amide protons in WT-HlyIIC that give resolved peaks for the conformers related by *cis/trans* isomerization of P405 (the *cis* form resonances are indicated with green labels in Fig. 5A). The *cis* resonances are not seen in the P405M mutant which only has the *trans* form. Because *cis/trans* isomerization occurs on a much faster timescale than HX, the HX rates for the *cis* and *trans* forms are very similar and only the protection factors for the *trans* form of WT-HlyIIC are given in Fig. 5B. Protection factors are mapped on the NMR structure for WT-HlyIIC (Fig. 5D) and P405M-HlyIIC (Fig. 5E).

Backbone amide proton protection factors in WT-HlyIIC are uniformly large for residues in regular secondary structure. The exception is helix αA, where only a single residue shows weak protection (Fig. 5B,D). In the P405M mutant (Fig. 5C), the largest protection factors are reduced about 2.5-fold compared to WT, consistent with the lower stability of the mutant to unfolding (Fig. 3B). A much more dramatic change is seen when the structure distribution of protection factors in the P405M mutant is considered. The amide protons from subdomain 2 in the second half of the molecule are protected but only two amide protons from subdomain 1 show measurable protection, at the beginning of strand β2 (Fig. 5C,E). The two weakly protected amide protons from subdomain 1 exchange within 2 h of dissolving the protein in D_2_O (Fig. 5A). Moreover, within subdomain 2, the protection factors for helix αB are about 10-fold weaker than those from the β3-β4-β5 component of the structure (Fig. 5C). Taken together, these observations suggest that the P405M mutant, which is necessary to eliminate the *cis* proline conformation, not only globally destabilizes the HlyIIC domain by 5 °C compared to the WT, but also causes an uncoupling of the HX dynamics of subdomain 1 from subdomain 2. To a lesser extent, within subdomain 2 the dynamics of helix αB are also uncoupled from those of the β5-β3-β4 sheet. In addition to the backbone amide protons, the side-chain indole nitrogen proton of W372 from subdomain 2 is protected in both WT and the P405M mutant (not shown). In the NMR structure, the indole nitrogen proton of W372 forms an H-bond with the side-chain carboxylic acid group of E386 from subdomain 2, which in turn forms a salt-bridge to K342 from subdomain 1 (Fig. S1B).

The uncoupling of the P405M mutant structure into two subdomains may be related to the observation that this protein forms a folded dimer with well-dispersed NMR signals at protein concentrations above ~0.5 mM and temperatures above ~20 °C^25^. The conditions for the present study (0.4 mM protein concentration and a temperature of 15 °C) were chosen so as to avoid the dimeric form. In contrast to the P405M-HlyIIC mutant, the more cooperatively folded WT-HlyIIC shows no signs of oligomerization at protein concentrations as high as 2 mM and temperatures as high as 40 °C. Additional support for a weaker structural cooperativity in the P405M mutant comes from the thermal denaturation data in Fig. 3B, where the slope of the thermal unfolding curve for P405M-HlyIIC is shallower than that for the WT, a hallmark of a less cooperative folding transition.

### Modeling of the HlyIIC domain in the context of full-length HlyII

**Figure 6:**
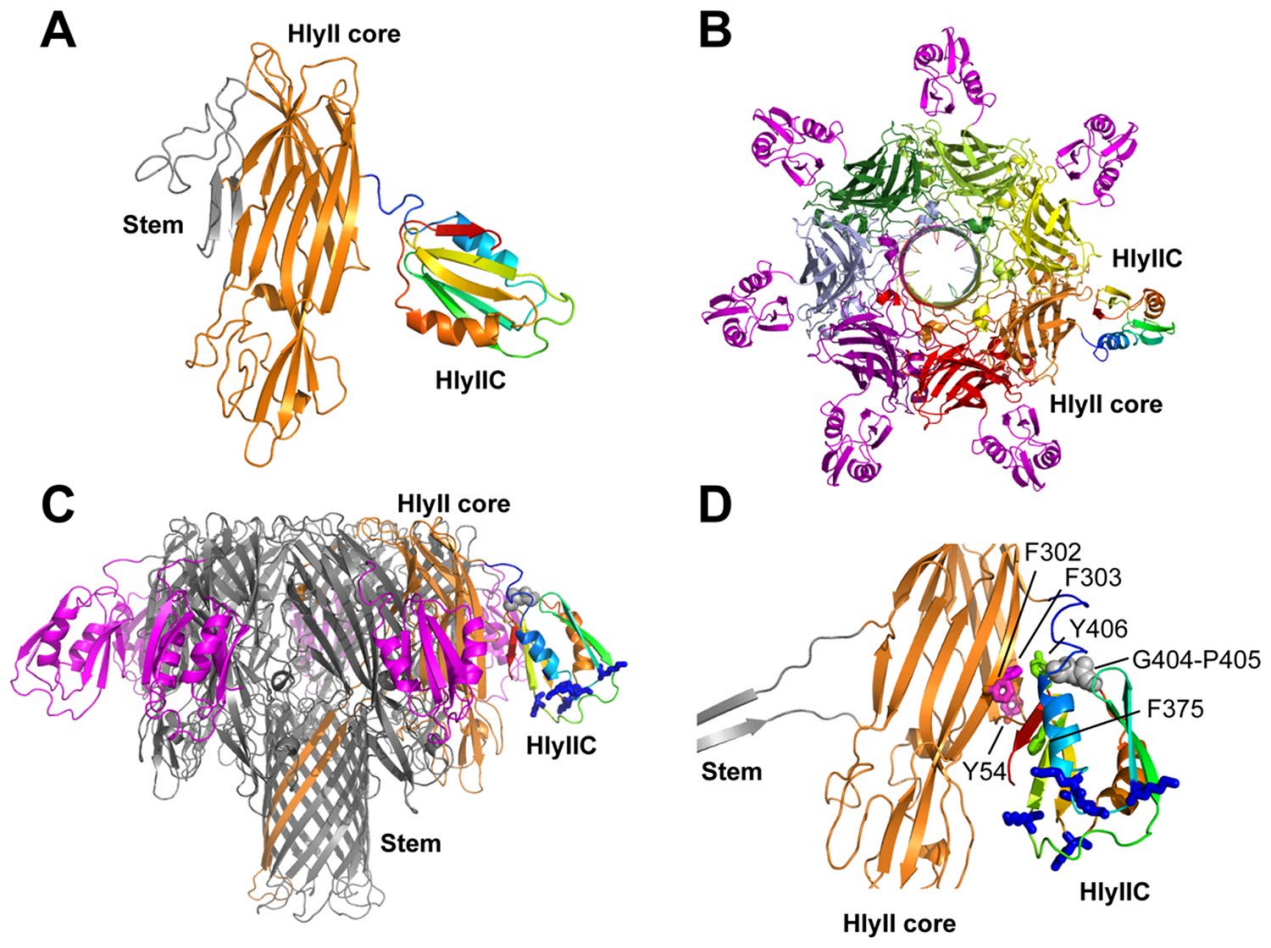
Modeling of full-length HlyII. (**A**) Model of the HlyII monomer based on the X-ray structure of monomeric *S*. *aureus* α-hemolysin (PDB code 4IDJ). The stem loop, which forms the trans-membrane β-barrel in the heptameric pore is colored gray, the core structure of HlyII in orange, and the HlyIIC domain in rainbow colors. (**B**) Model of the HlyII pore based on the X-ray structure of heptameric *S*. *aureus* α-hemolysin (PDB code 7AHL). The view is down the axis of the pore from the extracellular side, perpendicular to the plane of the membrane lipid bilayer. (**C**) View of the HlyII pore parallel to the plane of the lipid bilayer. The pore has a mushroom like structure, with the membrane-traversing β-barrel formed from the stem loops of seven monomers. The mushroom cap consists of the core domains. A protomer of the heptamer is colored in orange. In the model of the HlyII pore, the HlyIIC domain (shown in rainbow colors for one of the protomers) extends from the mushroom cap towards the membrane. (**D**) Expansion of the view in (**C**). The lysines that make up the positively charged patch of the HlyIIC domain are shown as blue sticks. The G404-P405 dipeptide, in a loop adjacent to the N-terminal connector, is depicted by gray spheres. Exposed aromatic residues that may dock the HlyIIC domain to the HlyII-core are labeled in the expansion.

To better understand the role of the HlyIIC domain in the context of the full-length HlyII toxin we calculated homology models of the latter (Fig. 6). The HlyII-core was modeled using the monomer (PDB 4IDJ) and protomer (7AHL) X-ray structures of the homologous toxin α-hemolysin from *S*. *aureus* with the SWISS-MODEL server^36^. The non-conserved HlyIIC domain, which is unique to HlyII, was linked and docked to the respective core structures with the *A*b *I*nitio *D*omain *A*ssembly (AIDA) server^37^. The AIDA program is used to predict the orientations of domains in multi-domain proteins^37^. The structure of each individual domain is treated as invariant, and only the structure of the linker between the domains is varied to energy-minimize an *ab initio* folding potential that seeks to optimize docking of the domains^37^. A model of the membrane-spanning pore structure was constructed by superposing the HlyII-core structure of the protomer calculated with the AIDA program onto each of the seven protomers in the 7AHL heptamer structure (Fig. 6B,C). An initial model without distance restraints resulted in steric overlap between the HlyIIC domain and the adjacent protomer that was not included in the docking simulation (Fig. S5). Noticing there were exposed aromatic residues in the HlyII-core structure (Y54, F302, F303) positioned to interact with exposed aromatic residues in the HlyIIC domain (F375,Y406), we set six distance restraints of 10 Å between the aromatic residues to obtain a revised final model (Fig. 6B–D). The orientations of the HlyII-core and HlyIIC domain were similar to the original model (Fig. S5), but in the revised model there were no longer steric clashes between the protomers. Of the aromatic residues, Y54 in the HlyII core, and F375 and Y406 in the HlyIIC domain appear to be conserved. The two residues F302 and F303 are conserved in sequence homologs that have the HlyIIC domain, but not in those where it is absent, suggesting a possible role of these aromatic residues in docking HlyIIC against the HlyII core.

## Discussion

The present work demonstrates that HlyII contains a structurally unique C-terminal domain, not present on PFTs that otherwise share structural homology and similar function. Indeed, the structure of HlyIIC, which consists of two β-sheets flanked by two α-helices in a barrel-like structure, represents a novel fold with no identifiable structural homologs. Because the HlyIIC structure represents a novel fold we anticipate it will shed light on structure-function relationships of yet to be discovered sequence and structure homologs.

A BLAST search of the HlyIIC sequence revealed that besides other proteins in the HlyII family, the closest sequence homologs are two gene fragments of unknown function found in *B*. *anthracis*. The first fragment corresponding to residues 319–371 in the N-terminal subdomain 1 of HlyIIC, occurs on the chromosome of the Sterne strain of *B*. *anthracis* (sequence ID: AAT54665.1, 87% sequence identity). The second fragment corresponding to residues 364–412 in the C-terminal subdomain 2 of HlyIIC, is found in the XO1 plasmid (sequence ID: WP_010890031.1, 38% sequence identity)^12, 38^. The XO1 plasmid, together with XO2, is needed for anthrax toxicity.

Because the HlyIIC domain exists in a dynamic equilibrium due to *cis/trans* isomerization of P405, we solved the NMR structure of the proline to methionine single point mutant P405M-HlyIIC. CD data (Fig. 3A), as well as the similarity of NMR chemical shifts between the WT and the mutant^25^, indicate that the structure of WT-HlyIIC is very similar to the P405M-HlyIIC mutant. A notable difference is that strand β5 is extended by three to four residues in the *cis* compared to the *trans* form of WT-HlyIIC, based on a dihedral angle analysis of backbone NMR chemical shifts^25^.

The P405M mutation destabilizes the HlyIIC domain by 5 °C to thermal denaturation (Fig. 3B) and leads to an uncoupling of the dynamics of subdomain 1 from subdomain 2 (Fig. 5B–E). That the two subdomains have properties of independent folding units and the observation that the subdomains have homology to separate gene fragments in *B*. *anthracis*, suggests that the HlyIIC domain may have arisen through a gene fusion event. If the HlyIIC domain originated from the fusion of subdomain 1 (αA-β1-β2) with subdomain 2 (β3-β4-αB-β5), evolutionary pressure would have favored the accumulation of mutations that maximized the folding cooperativity of the structure. Because it is destabilizing, the P405M mutation could relieve the requirement for maximum folding cooperativity, and reveal the original bipartite organization of the structure into two subdomains with partially autonomous folding properties. A similar mechanism has been proposed for the organization of subdomains into cooperatively folded structures in the OB-fold family of proteins^29^. Conversely, the two subdomains encoded on separate genes in *B. anthracis﻿*, could come together through fragment complexation to form a protein complex with a similar structure to the HlyIIC domain.

The function of the HlyIIC domain is currently unknown. An earlier structural model for HlyIIC was proposed^21^ based on a sequence to structure *ab initio* prediction using the program ROSETTA, although the convergence of the predicted structures was poor^21^. Based on this model it was suggested that the HlyIIC domain had a structure similar to the iron-binding protein frataxin, and a possible iron-binding function^39^. While the two proteins have similar secondary structure elements, the β-sheet topology of HlyIIC is different from frataxin, where the β-strand connections are all sequential and the last α-helix follows the last β-strand. Frataxin forms a flat continuous β-sheet with the two α-helices on one side of the sheet, while HlyIIC forms a barrel-like structure. The coordinate RMSD between the NMR structure of HlyIIC and frataxin (PDB code 1EKG) is ~14 Å, indicating quite different structures.

Because many PFTs contain accessory domains that are carbohydrate-binding lectins^40^, we submitted the HlyIIC domain to a glycan array screen run by the Consortium for Functional Glycomics (CFG) (www.functionalglycomics.org). Screening a library of over 600 mammalian glycans failed to identify any hits for HlyIIC (CFG accession code: primscreen_5868). Although this negative result suggests that HlyIIC is not a carbohydrate-binding protein, it does not rule out this possibility. First, the HlyIIC domain on its own, may have a weaker affinity for carbohydrates than when part of the PFT formed from full-length HlyII. Second, the *B*. *cereus* HlyII toxin and HlyIIC domain are also found in *B*. *thuringiensis*, a bacterium that infects insects. If HlyIIC binds carbohydrates specific to insects, these may have been missed in the mammalian glycan library.

To further explore how the HlyIIC domain interacts with the rest of the toxin, we modeled its NMR structure in the context of full-length HlyII (Fig. 6). Without functional data on the full-length HlyII toxin, which is difficult to express, such modeling is speculative but could provide clues about possible roles for HlyIIC based on its position in the overall structure. We wondered if the HlyIIC domain could displace the stem loop (gray in Fig. 6A) in the monomer structure, so as to favor the membrane-bound protomer conformation of HlyII, in which the stem loop participates in the heptameric β-barrel pore. In the monomer model (Fig. 6A), the HlyIIC domain extends from the toxin core at an edge of the β-sandwich structure opposite that of the stem loop, so that an interaction between the two segments is highly unlikely. Fig. 6B and C show the homology model of the heptameric membrane-spanning pore complex. Even with a fully extended linker, the domain appears to be too far away to interact with the pore component of the structure (Fig. 6B and C), in agreement with experimental results that indicate HlyIIC does not obstruct the toxin pore in voltage gating experiments^12^. Rather, the HlyIIC domain in the heptamer model is positioned towards the membrane with the positively charged patch formed by the lysine residues at the bottom of the structure poised to interact with negatively charged molecules such as lipids carbohydrates, or proteins embedded in the membrane (Fig. 6C). Biochemical studies showed that HlyII with HlyIIC deleted, can still form oligomeric pores that have only an 8-fold reduction in activity compared to the full-length wild type^12^. Oligomers of the toxin without the HlyIIC domain were less stable in SDS than those with the domain, the former dissociating at 78 °C compared to 82 °C for the latter^12^. The HlyII toxin is relatively non-specific being able to lyse a variety of eukaryotic cell types^13, 14^, as well as being able to form active pores in model membrane systems such as liposomes without the C-terminal HlyIIC domain^21^. Thus, putative membrane interactions of the positively-charged HlyIIC surface with negatively-charged lipid, carbohydrate, or protein moieties on cell membranes could afford a general mechanism for cell targeting or stabilization of the PFT complex in the membrane.

An interesting question regards the role played by *cis/trans* isomerization of P405 in the HlyII toxin. Splitting of backbone ^1^H-^15^N signals from sites distributed throughout the structure is evident in the ^1^H-^15^N HSQC spectrum of the HlyIIC domain (Fig. 2D), so that binding events at distal sites could be communicated to P405. For example, the proposed binding of the positively charged patch of HlyIIC to negatively charged moieties in the membrane could affect the ratio of *cis* to *trans* isomers for P405, located in a loop between strands β4 and β5 that is directly adjacent to the N-terminal linker that connects the HlyIIC to the HlyII-core (Fig. 6D). The linker segment between the N-terminus of HlyIIC domain and the C-terminus of the HlyII core is highly flexible and can be proteolytically cleaved^12, 21^, making it unlikely to affect communication between the two domains. However, P405 immediately precedes the conserved exposed aromatic residue Y406 in HlyIIC, that may form contacts with aromatic residues from the HlyII-core (Fig. 6D). A considerably different alternative role for the proline, is that it could constitute a binding site for cyclophilin. The site of proline isomerization in HlyIIC, P405, is part of the sequence 404-GPYI-407, which is close to the consensus sequence GPxL recognized by cyclophilins^41, 42^. Moreover, cyclophilin-binding should favor prolines undergoing *cis/trans* isomerization^43^, such as P405 in HlyIIC. It has recently been reported that leukotoxin binds to cyclophilin D in neutrophil mitochondria thereby triggering the disruption of the mitochondrial membrane potential, release of cytochrome c, and apoptosis^44, 45^. The induction of apoptosis in neutrophils may help bacteria evade immune defenses. It remains to be seen whether HlyII can interact with mitochondrial cyclophilin D, and serve a similar role.

In conclusion, the structure of the HlyIIC domain represents a new fold in which two subdomains each comprised of an α-helix and a β-sheet (αA-β1-β2 and β3-β4-αB-β5) come together to form a barrel-like structure, stabilized by a core formed from three layers of hydrophobic residues. The different dynamic properties of the two subdomains when the HlyIIC domain is destabilized by the P405M mutation, together with the existence of two homologous gene fragments in *B*. *anthracis*, suggests that the HlyIIC domain may have arisen from a genetic fusion of the two subdomains. The most likely function for the HlyIIC domain is to stabilize the heptameric PFT, either through interactions with adjacent protomers in the heptamer or through membrane binding. The unique proline P405 in the HlyIIC domain, which exists in two conformational states due to *cis/trans* isomerization, could act as a conduit to communicate binding events to the core of the HlyII toxin or could constitute a binding site for cyclophilin D. The structure and dynamics of HlyIIC characterized in this work pave the way for experiments that can better establish the function of this novel accessory domain in PFTs.

## Methods

### Expression and purification of HlyIIC

The HlyIIC domain P405M mutant (P405M-HlyIIC) was expressed and purified as described previously^25^ and isotopically enriched with ^15^N and ^13^C using established protocols^46^. Triple-labeled samples (^15^N^13^,C, and ^2^H) were produced by slowly acclimatizing *E*. *coli* to grow in deuterium oxide (D_2_O) by incremental amounts^47^. Briefly, T7 Express cells (New England Biolabs Inc., Ipswich, MA) containing the HlyIIC gene fragment (residues D319-I412) cloned into the *pET28b* plasmid, were grown overnight at 37 °C in M9 minimal media supplemented with 30 μg/ml kanamycin using glucose (3 g/L) as a carbon source. A 10 ml culture was inoculated with 500 μL of the overnight culture, and grown to mid-log phase (OD_600_ = 0.6–0.8) with vigorous aeration. This culture was used to inoculate new M9 media containing 30% D_2_O and grown to the same OD as the first. In a similar fashion, each culture was used to inoculate fresh media with successively increasing amounts of D_2_O (0%, 30%, 60%, 90%, 100%). The final culture (500 ml) contained 1.5 g/l ^15^N-labeled NH_4_Cl and 3 g/l ^2^H, ^13^C-enriched glucose in D_2_O. Protein was expressed for 18 hours at 37 °C and purified by Ni-NTA affinity and size-exclusion chromatography as described previously^25^. The His_6_-affinity tag was removed by cleavage with a 1:100 wt/wt ratio of thrombin for 1 h at room temperature (Haemotologic Technologies Inc., Essex Junction, VT).

### NMR structure determination

P405M-HlyIIC samples for NMR were at a concentration of 0.4 mM in 20 mM NaH_2_PO_4_ buffer, pH 6.1. All NMR work on the mutant was done at a temperature of 15 °C to suppress signals from a dimeric form unique to the P405M-HlyIIC mutant, which is stabilized at large protein concentrations and high temperature^25^. NMR spectra were recorded using an 800 MHz Varian Inova Spectrometer equipped with a cryogenic probe. Chemical shift assignments for P405M-HlyIIC and the *cis* and *trans* forms of WT-HlyIIC were published previously^25^. NOE-based distance restraints were obtained from 3D ^15^N- and ^13^C-NOESY experiments with mixing times of 125 ms. These were supplemented with hydrogen bond restraints from a long-range HNCO (lrHNCO) experiment that identifies hydrogen bond acceptors and donors via through-H-bond couplings^28^. The lrHNCO experiment was recorded in TROSY mode on a ^2^H, ^13^C, ^15^N triple-labeled sample of HlyIIC. Backbone (ϕ, ψ) and sidechain (χ1) torsion angles were calculated from HN, Hα, Cα, Cβ, CO, and N chemical shifts using the program TALOS-N^48^. Stereospecific assignments for methylene protons were determined from a 3D HNHB spectrum, 50 ms mixing time 2D NOESY spectra, and ^1^H-^15^N NOESY-HSQC experiments^49^. NMR structures of P405M-HlyIIC were calculated from 1736 experimental restraints (Table 1) using the programs X-PLOR NIH 2.36^50^ and ARIA 2.3^51^. In the final optimization step, structures from X-PLOR NIH were refined in water with ARIA. The 25 lowest energy NMR structures have been deposited in the Protein Data Bank under the PDB accession code 2N67.

### Backbone dynamics from NMR relaxation experiments

Backbone dynamics of P405M-HlyIIC were investigated using ^15^N longitudinal (R1), transverse (R2), and cross-relaxation (^1^H-^15^N NOE) experiments. All relaxation data were acquired at a field strength of 800 MHz. R1 rates were obtained using interleaved relaxation delays of 0.05, 0.13, 0.21, 0.49, 0.57, 0.71 and 0.99 s. R2 rates were determined using interleaved relaxation delays of 0.01, 0.03, 0.05, 0.07, 0.09, 0.11, 0.15, 0.25 s. A 2 s pre-acquisition delay was used for recovery to thermal equilibrium. ^1^H-^15^N NOE values were determined from the ratio of crosspeak intensities in a spectrum for which the proton signals were saturated for 4 s and a control spectrum in which the saturation period was replaced by a pre-acquisition delay of equivalent length. The processing and analysis of relaxation parameters was done according to published methods^52, 53^. Model-free analyses^34^ of the ^15^N relaxation data to obtain *S* ^2^ order parameters and R2_ex_ conformational-exchange terms were performed with the program Tensor2^54^, and yielded an optimal global rotational correlation time of 9.7 ns.

### Circular dichroism (CD)

HlyIIC-WT, HlyIIC-P405M, and HlyIIC-P405A were dialyzed into buffer containing 10 mM NaH_2_PO_4_, pH 7.5 and 50 mM NaCl, and diluted to a concentration of 4 μM. CD data were collected on a Jasco J-810 spectrometer (Jasco Inc., Easton, MD). Thermal denaturation was monitored using the change in molar ellipticity at 220 nm while changing the temperature in 5 degree increments from 20 °C to 90 °C. T_m_ values were calculated from the mid-point of sigmoidal fits to the temperature data.

### Hydrogen exchange experiments

Hydrogen exchange (HX) experiments on 0.4 mM samples of WT-HlyIIC and the P405M-HlyIIC mutant were performed on a 600 MHz spectrometer at pH 6.2 and a temperature of 10 °C. To initiate HX, lyophilized ^15^N-labeled samples were dissolved in 99.96% D_2_O and ^1^H-^15^N HSQC spectra were recorded as a function of time. Decays of ^1^H-^15^N crosspeak intensities with D_2_O incubation times were fitted to single exponentials, to obtain HX rate constants and their associated uncertainties^55^. Protection factors were calculated as the intrinsic HX rates divided by experimental HX rates. Intrinsic HX rates for the HlyIIC sequence were calculated using the SPHERE server (http://landing.foxchase.org/research/labs/roder/sphere/)^56^.

### Modeling of full-length HlyII

The core structure of HlyII was modeled using the homologous *S*. *aureus* α-hemolysin toxin with the Swiss-Model server (https://swissmodel.expasy.org)^36^. The NMR structure of the non-conserved HlyIIC domain was linked and docked to the homology model of the HlyII core structure with the *A*b *I*nitio *D*omain *A*ssembly (AIDA) server (http://ffas.sanfordburnham.org/AIDA/), a program used to predict the relative orientations of domains in multi-domain proteins^37^. A model of the heptameric membrane-spanning pore, was constructed by superposing the HlyII-core structure calculated with AIDA onto each of the seven protomers in the α-hemolysin structure.

### Data availability

The atomic coordinates and restraint files for HlyIIC-P405M are deposited in the PDB with accession code 2N67. Chemical shifts are deposited under BMRB entries 19463 (HlyIIC-P405M), 19462 (*trans* WT-HlyIIC), and 19461 (*cis* WT-HlyIIC). Additional datasets if not included in this published article (and its Supplementary Information Files) are available from the corresponding author on reasonable request.

## Electronic supplementary material

Supporting Material
