# Supplementary material for: NMR structure of the Bacillus cereus hemolysin II C-terminal domain reveals a novel fold
Source: Sci Rep. 2017 Jun 12;7:3277. doi: 10.1038/s41598-017-02917-4 (PMC5468326; doi:10.1038/s41598-017-02917-4)
Supplement: Supplementary file 1 — Supporting Material [file 41598_2017_2917_MOESM1_ESM.pdf]

Supporting Material for

NMR structure of the *Bacillus cereus* hemolysin II C-terminal domain reveals a novel fold

Anne R. Kaplan[1], Katherine Kaus[2], Swastik De[2,3], Rich Olson[2], Andrei T. Alexandrescu[1]**

[1]Department of Molecular and Cell Biology, University of Connecticut, Storrs, CT, 06269, USA

[2]Department of Molecular Biology and Biochemistry, Wesleyan University, Middletown, CT, 06459, USA

[3]Present address: Department of Molecular Biophysics and Biochemistry, Yale University, 266 Whitney Avenue, New Haven, CT, 06520-8114

*Corresponding authors: Andrei Alexandrescu, Department of Molecular and Cell Biology, University of Connecticut, 91 N. Eagleville Rd., Storrs, CT 06269-3125, Tel: 860-486-4414, Fax: 860-486-4331, E-mail: andrei@uconn.edu; Rich Olson, Department of Molecular Biology and Biochemistry, Wesleyan University, 224 Hall-Atwater, 52 Lawn Ave., Middletown, CT 06459-0175, Tel: 860-685-3070, Fax: 860-685-2141, E-mail: rolson@wesleyan.edu

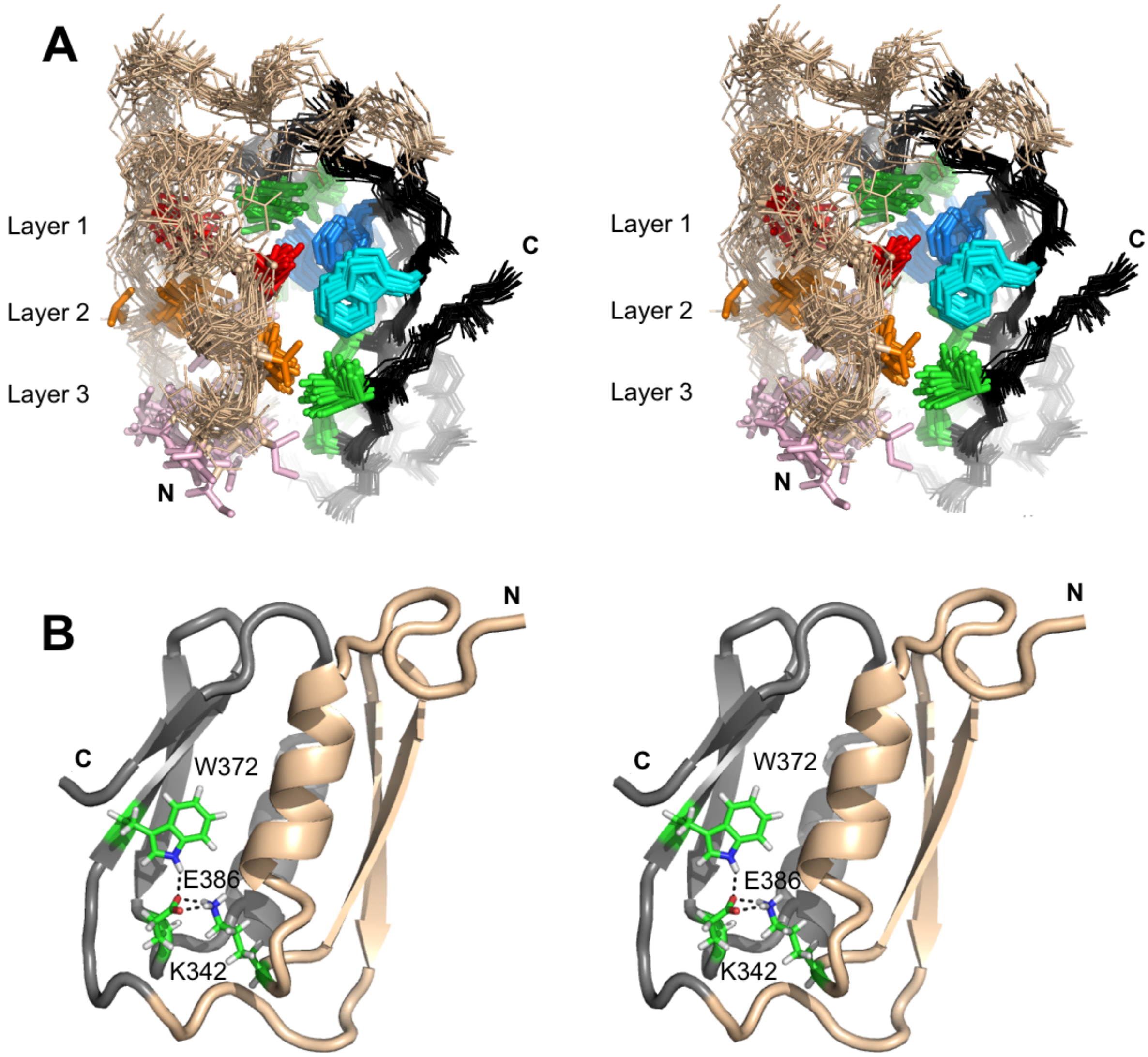

Supplementary Figure S1 Stereo-pair diagrams ('cross-eyed' view) illustrating side-chain interactions in HlyIIC. (**A**) Precision of side-chains in the ensemble of top 25 NMR structures for P405M-HlyIIC. The figure was obtained by superposing the NMR ensemble heavy atoms of residues 330-412 onto their mean coordinates. The backbone is colored peach for residues 330-368 and gray for residues 369-412.The three-layer structure of the hydrophobic core is illustrated. Hydrophobic residues from the first half of the molecule (330-368) are colored in red, orange and pink. Hydrophobic residues from the second half of the molecule (369-412) are colored in blue, cyan (aromatic residues) and green (aliphatic residues). Note that the precision of both the backbone and side-chains are better for the C-terminal half of the protein. The residues that contribute to the three-layer hydrophobic core are as follows. Layer 1: L338 (αA), L345 (β1), W372 (β3), F384 (β4), V390 (αB), I393 (αB), I412 (β5); Layer 2: V334 (αA), L347 (β1), A356 (β2), I374 (β3), Y382 (β4), I397 (αB), I409 (β5); Layer 3: I331 (αA), M349 (β1), L354 (β2), V376 (β3), V381 (β4), L400 (αB), L402 (αB), I407 (β5). (**B**) H-bond interactions involving W372 illustrated with the NMR structure closest to the ensemble average. The W372 Nε1 side-chain indole proton is protected from solvent exchange and is H-bonded to the side-chain carbonyl of E386, which also forms a H-bonded salt-bridge to K342.

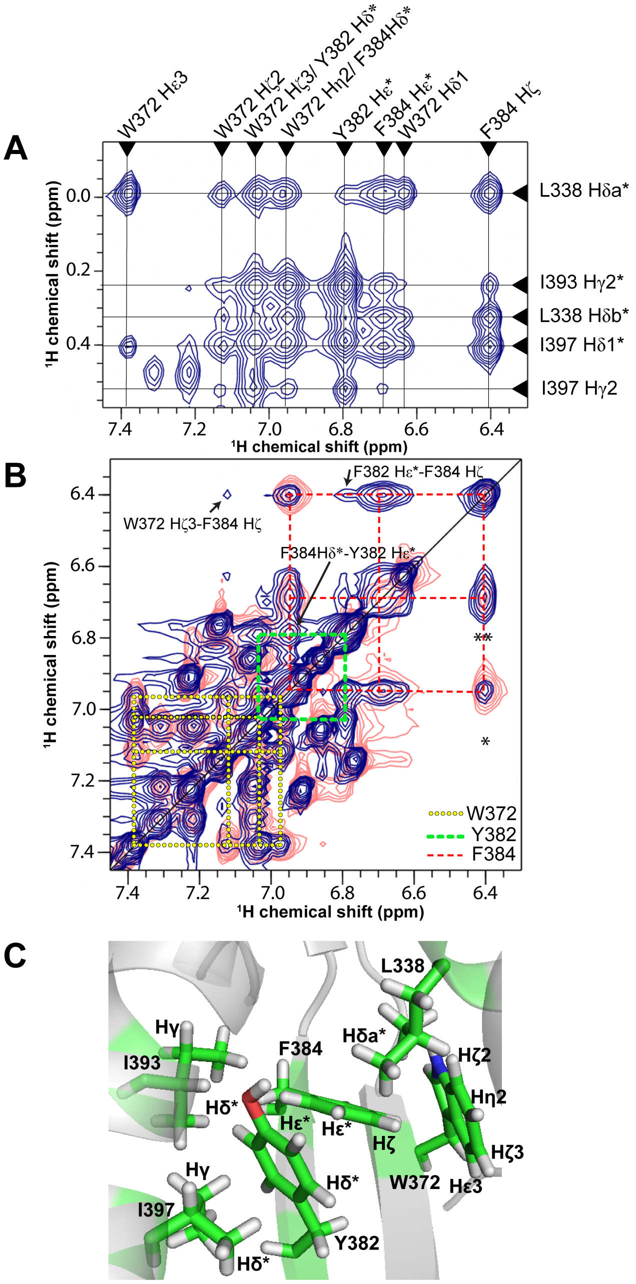

Supplementary Figure S2 The fold of HlyIIC is buttressed by hydrophobic residue interactions between the β-sheets and flanking α-helices. Methyl (**A**) and aromatic (**B**) portions of a 2D ^{1}H-NOESY spectrum of HlyIIC-P405M showing some of these interactions. In (A) the aromatic residues W372, Y382, and F384 from the 5-3-4 β-sheet contact the upfield shifted Hδ methyl groups of L338 from helix αA, and the γ and δ methyl groups of Ile393 and Ile397 from helix αB. **B.** Overlay of the aromatic portions of 2D TOCSY (pink) and 2D NOESY(navy) spectra. The aromatic ring spin-systems of W372, Y382, and F384 are traced out with dotted lines in the indicated colors. The W372 Hδ1 resonance was assigned from an NOE to the Nε1 proton at 12 ppm (not shown). Arrows denote NOEs between aromatic residues, where the symbols "*" and "**" indicate weak crosspeaks, symmetrically-related about the diagonal, that are only seen at lower contour levels than shown. **C.** The network of hydrophobic residues in the HlyIIC-P405M NMR structure giving rise to the NOEs in (A) and (B).

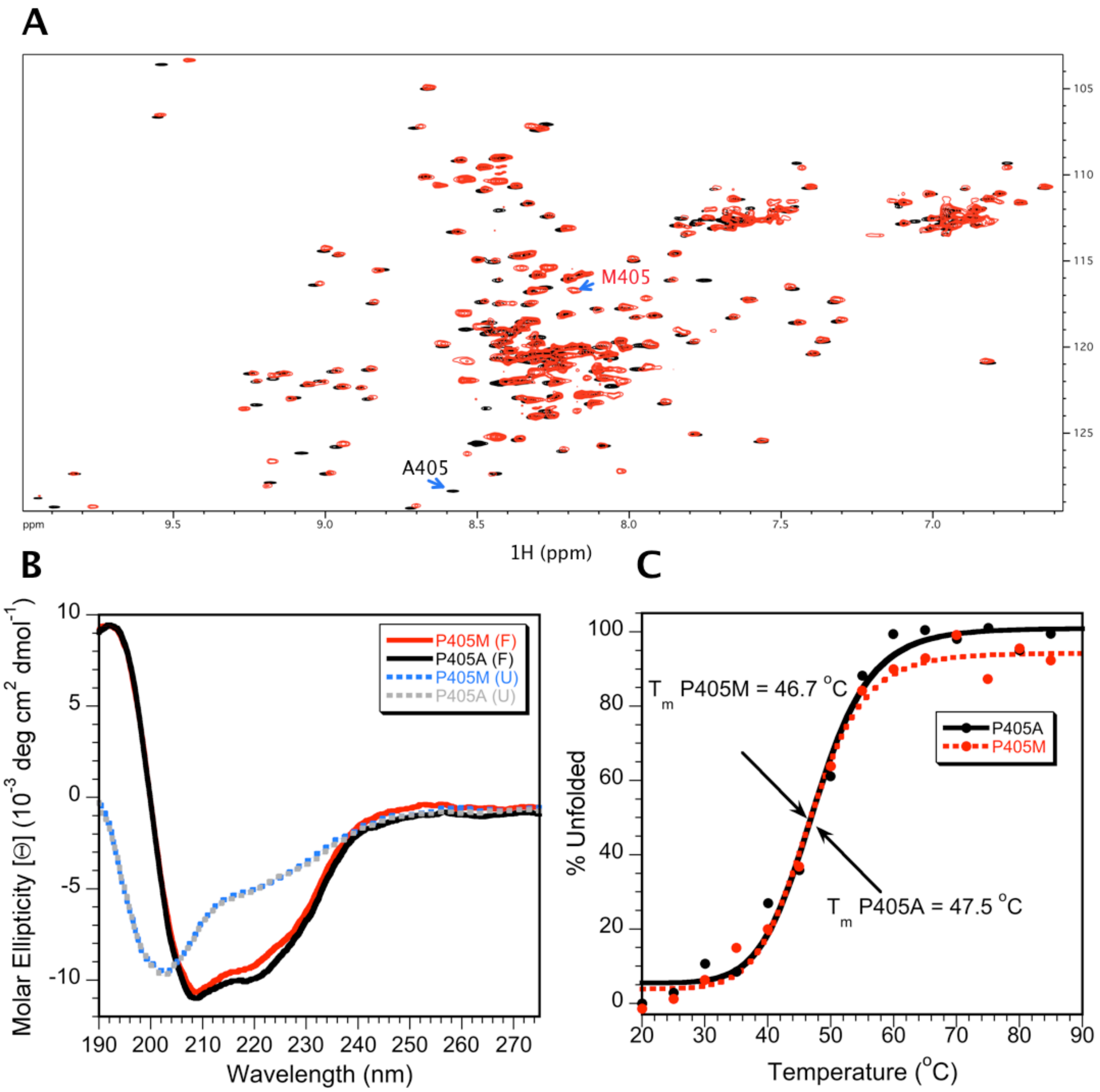

Supplementary Figure S3 Comparisons of the P405M and P405A mutants that replace P405 in HlyIIC, and stabilize the *trans* state. (**A**) Superposition of the ^{1}H-^{15}N HSQC spectra for P405A-HlyIIC (black) and P405M-HlyIIC (red) highlighting the similarity of the spectra. The assignment for M405 in P405M-HlyIIC (blue arrow) is based on 3D NMR spectra while that for A405 in P405A-HlyIIC is tentative. (**B**) Circular dichroism wavelength scans for the folded mutants at 20 °C (solid lines) and the thermally unfolded mutants at 90 °C (dotted lines). (**C**) Thermal unfolding of P405A-HlyIIC (black) and P405A-HlyIIC (red). The circular dichroism data for P405M-HlyIIC is the same as in Fig. 3, and is repeated here for comparison with P405A-HlyIIC.

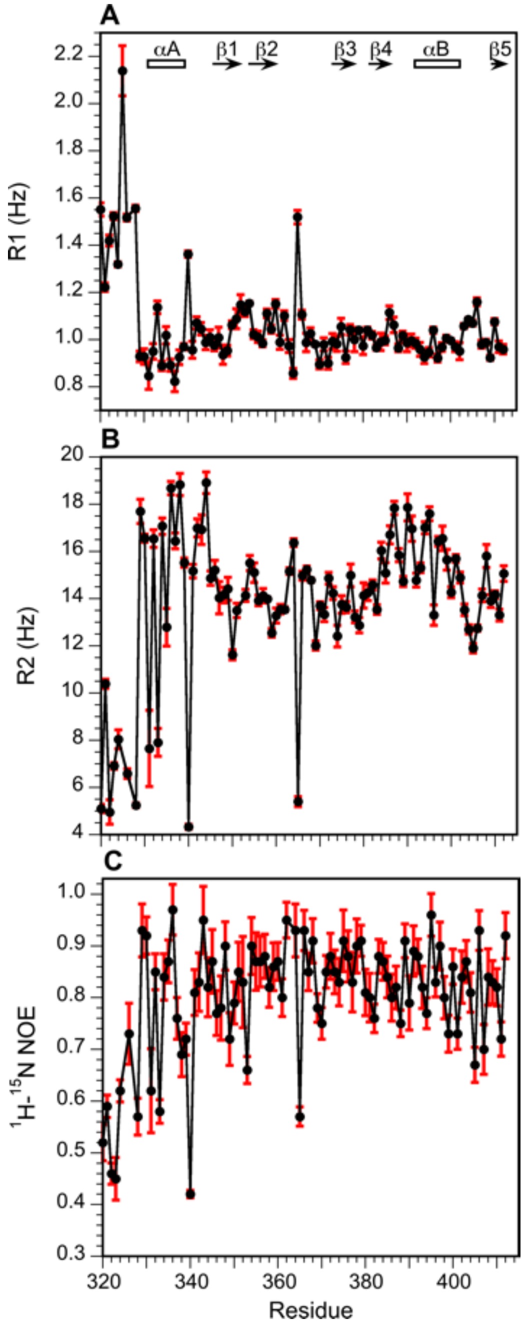

Supplementary Figure S4 ^{15}N-NMR relaxation data for the HlyIIC domain. (**A**) R1, (**B**) R2, and (**C**) $^{1}H-^{15}N$ NOE values. The secondary structure of the domain is shown in the inset to panel A.

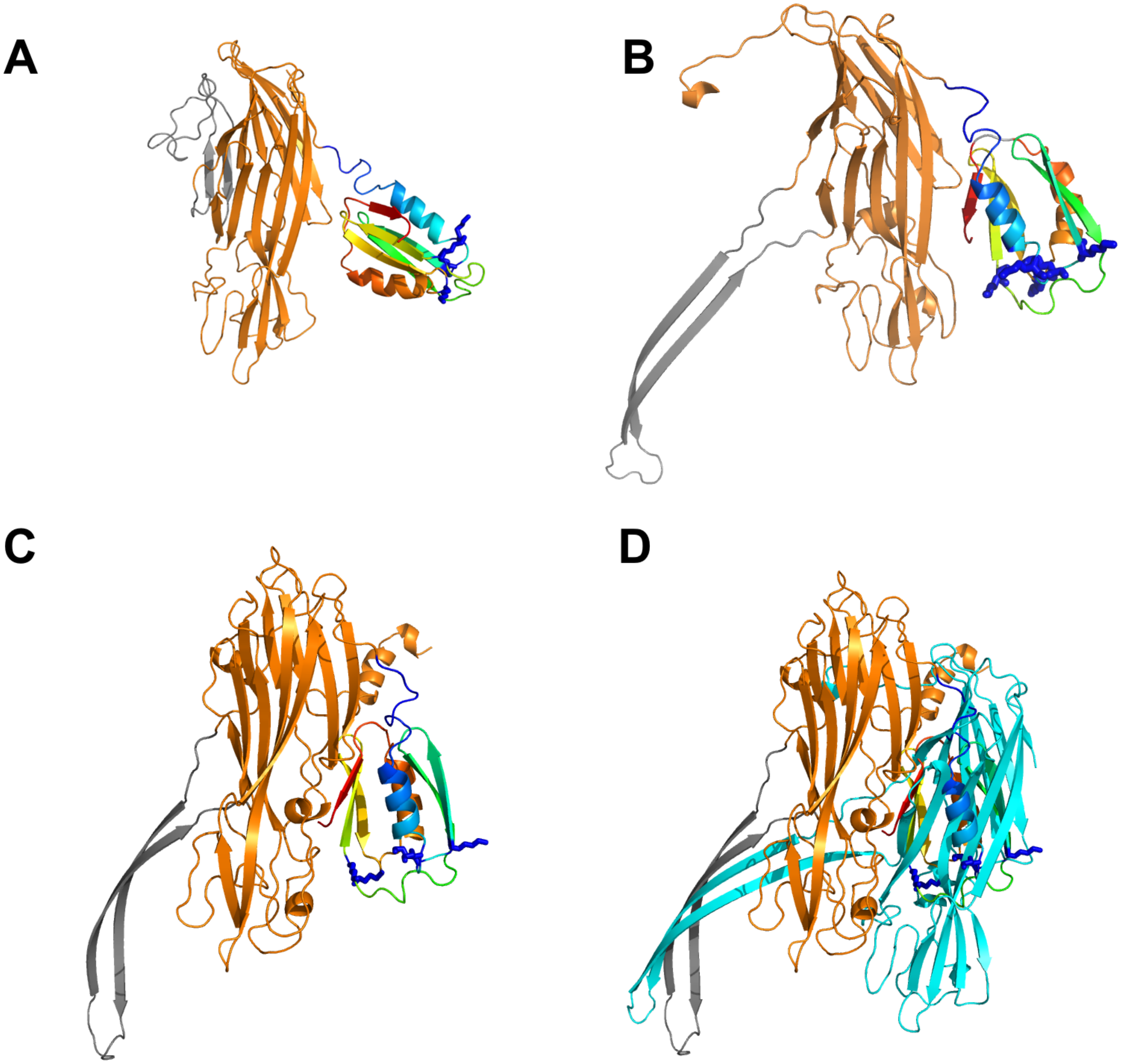

Supplementary Figure S5 Models of full-length HlyII. (**A**) The HlyII monomer. The HlyII-core structure is shown in orange, the HlyIIC domain in rainbow colors, and the stem loop which folds up against the HlyII-core structure in the monomer, is show in gray. (**B**) The HlyII protomer of the heptameric pore complex. The model was obtained using distance constraints of 10 Å between the aromatic residues Y54, F302, F303 of the HlyII-core structure, and F375, Y406 of the HlyIIC domain. This is the same model as shown in Fig. 6B-C of the main text. (**C**) Model of the HlyIIC protomer without any distance constraints. The orientation of the HlyIIC domain is similar to that in (B), with the lysines in the same plane as the top of the membrane-embedded stem loop (gray). (**D**) In contrast to the model in B, the protomer model without distance restraints leads to steric occlusion between the HlyIIC domain and the HlyII-core structure of the adjacent protomer in the heptamer (cyan).
